# Viburnum Prunifolium in Uterine Diseases

**Published:** 1882-03

**Authors:** 


### VIBURNUM PRUNIFOLIUM IN UTERINE DISEASES.

Dr. E. C. Mann (Boston Med. and Surg. Journal), states the following in reference to the use of this remedy: It appears to him to act directly and specifically upon the special nerves of the uterus as a true nerve sedative. He has had several very violent cases of congestive and neuralgic forms of dysmenorrhea, being accompanied by epileptiform convulsions of a very severe type, and in each and every case he has seen almost magical relief following the use of the fluid extract of viburnum prunifolium. The case referred to which was so severe that the intensity of the pain had worn out the unhappy sufferer and induced the epileptiform attacks, was completely cured in a few weeks by the combined use of the viburnum prunifolium and the use of the constant current of electricity; the positive pole being applied to the hypogastric region, and the negative pole, to which was attached a cup-shaped electrode, directly to the uterus. . . . . . He had failed to perceive any action on the general system, the whole force of the medicine appearing to be directed to the uterus and its system of nerves. When the pulse has been high from nervous excitement, and the temperature-centres in the brain have been temporarily paralyzed, allowing sudden rise in temperature, from nervous excitement, both pulse and temperature have fallen to the normal as the uterine pain has been relieved. His cases have been aggravated ones, many of them have been sent to Sunnyside on the verge of insanity.

